# Case Report: Multiple Vertebral Compression Fractures in 14-Year-Old Children With Multiple Myeloma

**DOI:** 10.3389/fonc.2021.662169

**Published:** 2021-03-25

**Authors:** Xia Wang, He He, Mei Zhang, Chuan Li, Chengyao Jia

**Affiliations:** ^1^Department of Laboratory Medicine, West China Hospital, Sichuan University, Chengdu, China; ^2^Department of Thoracic Surgery, West China Hospital, Sichuan University, Chengdu, China

**Keywords:** multiple myeloma, adolescent patient, spine compression fracture, autologous hematopoietic stem cell transplantation, monoclonal spike

## Abstract

Multiple myeloma (MM) is a neoplastic disorder characterized by clonal proliferation of malignant plasma cells derived from B cells in bone marrow. Pediatric MM is rare with only approximately 0.3% of cases diagnosed before the age of 30. In this report, we present a 14 years old boy diagnosed as MM with multiple pathologic vertebral fractures. To our knowledge, our patient is the youngest Chinese case in the literature to present with MM. He was treated with bortezomib, dexamethasone, and cyclophosphamide followed by autologous hematopoietic stem cell transplantation with good clinical response. We hope to aid in the understanding of the pathophysiology and management of this condition.

## Introduction

Multiple myeloma (MM) is a neoplastic disorder of B-cell origin caused by clonal proliferation of malignant plasma cells in bone marrow (1). The monoclonal protein, the malignant cells or cytokines secreted by the malignant proliferating myeloma cells lead to end-organ damage, such as bone destruction, pathologic fractures, renal insufficiency, anemia, and hypercalcemia (2). MM accounts for approximately 1% of all neoplastic diseases and the median age at diagnosis is approximately 70 years (3, 4). Pediatric MM is rare with only approximately 0.3% of cases diagnosed before the age of 30 (5). Given the low incidence of MM in children and adolescents, the clinicopathological characteristics and prognosis of these patients are not well known. However, the continuous implementation of novel agents against MM and autologous stem cell transplantation (ASCT) has likely led to better outcomes than standard therapy (6). ASCT has been a backbone treatment for fit MM patients over the last decades.

In this report, we describe a 14 years old boy diagnosed as MM with multiple pathologic vertebral fractures who was successfully treated with ASCT. To our best knowledge, this is the youngest Chinese case in the literature to present with MM (7).

## Case Report

**Figure 1:**
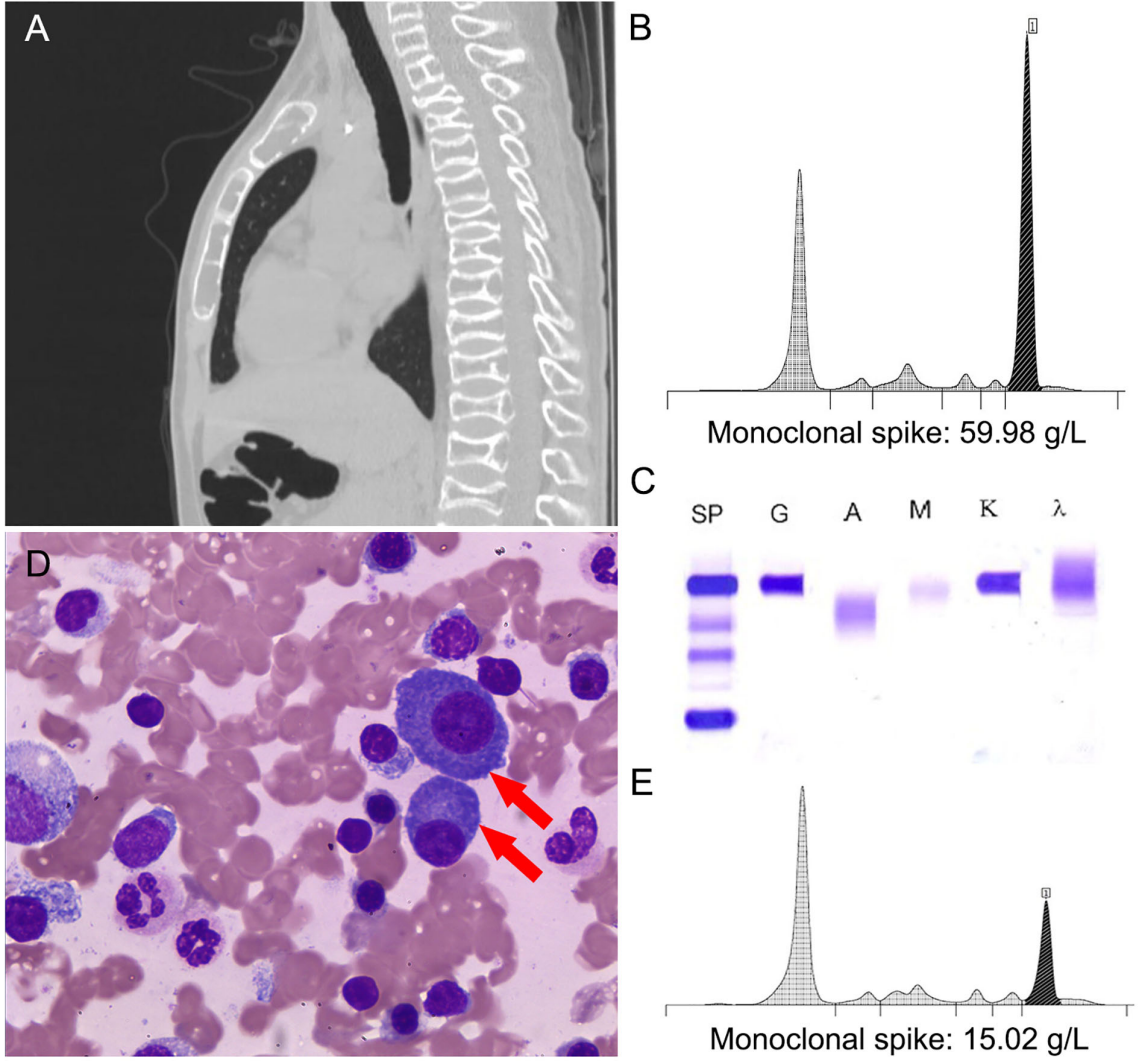
**(A)** Chest computed tomography (CT) scan. **(B)** Protein electrophoresis for gamma globulin levels (black) before treatment. **(C)** Serum immunofixation electrophoresis demonstrating monoclonal IgG kappa proteins. **(D)** Bone marrow cytology (red arrows: plasma cells, ×400). **(E)** Protein electrophoresis for gamma globulin levels (black) after autologous hematopoietic stem cell transplantation (ASCT).

An otherwise healthy 14 years old boy presented with a history of back pain that had been increasing for the previous month after an accidental fall. The chest X-ray images from previous hospital demonstrated multiple thoracic spine compression fractures in T5, T10, and T12. He was then referred to our hospital for further investigation. He had no significant family history. Chest computed tomography (CT) scan showed multiple compression fractures of the vertebral bodies (**Figure 1A**).The complete blood count showed normocytic anemia with a hemoglobin level of 101.0 g/L (130–175 g/L). Serum biochemistry showed total protein 126 g/L (65–85 g/L), albumin 32.3 g/L (40–55 g/L), whereas renal function, serum calcium, serum phosphorus and lactate dehydrogenase were normal. Further serum protein electrophoresis revealed monoclonal spike 59.98 g/L (**Figure 1B**). Immunofixation electrophoresis (**Figure 1C**) showed immunoglobulin G 85g/L (8–15.5 g/L), immunoglobulin A 308 mg/L (836–2,900 mg/L), immunoglobulin M 250 mg/L (700–2,200 mg/L), kappa light chain 83.50 g/L and lambda light chain 0.84 g/L. Serum β2 microglobulin 1.98 mg/L (0.7–1.8 mg/L). Bone marrow cytology showed plasma cells made up 6.5% of the population (**Figure 1D**). Flow cytometry of bone marrow showed the abnormal cells were CD38, CD138, CD56 and cKappa positive. Fluorescence *in situ* hybridization (FISH) for the gene myelocytomatosis oncogene was normal. Cytogenetics revealed a point mutation in exon 4 (c.215C>G: p.Pro72Arg) with a 46, XY karyotype. A diagnosis of multiple myeloma (IgG kappa, Durie–Salmon stage II) was made. Extramedullary plasmacytoma was not found.

The patient was then underwent induction chemotherapy with bortezomib (1.3 mg/m^2^ on days 1, 8, 15, and 22), cyclophosphamide (300 mg on day 1,8,15, and 22) and dexamethasone (15 mg on days 1–2, 8–9, 15–16, and 22–23) every three weeks for five cycles. After mobilization with granulocyte colony-stimulating factor, he proceeded to ASCT with a total number of mobilized CD34+ cells 1.58 × 10^6^/kg with melphalan (200 mg/m^2^) conditioning.

At the end of induction therapy, his back pain had subsided. The patient had a partial response according to the International Myeloma Work Group (IMWG) criteria. After ASCT, serum protein electrophoresis showed monoclonal spike 15.02 g/L. Serum biochemistry showed total protein 70.5 g/L (65–85 g/L). His monoclonal spike was reduced by 75% and he showed clinical improvement (**Figure 1E**). The patient received maintenance treatment with lenalidomide (25 mg/day) and his clinical condition was stable at 5-month follow-up.

## Discussion

MM is the second most common hematological malignancy after non-Hodgkin lymphoma (1–3). The median age at diagnosis of MM is nearly 70 years and it is very rare in juvenile with approximately thirty cases younger than 15 years (5, 8–13). In this article, we report a case of an extremely rare clinical presentation of MM in a 14-year-old boy with multiple pathologic fractures, which, to our best knowledge, is the youngest Chinese case in the literature to present with MM (7). Indeed, bone involvement, which occurs in almost 80% of patients with newly diagnosed disease, is the main cause of morbidity and mortality in MM patients (1). There have been previous sporadic reports of similar clinical presentations of MM in younger patients such as involving a mild inflammation in the T10 region in an 8-year-old boy, a destructive mass of the L3 vertebra in a 14-year-old boy, and a lytic bone lesion in the wing of the left ilium in a 17-year-old girl (9, 11, 12). In children and adolescents, although MM is rare in this population, prior reports showed a less aggressive clinical presentation than in adults (5, 13, 14).

The diagnosis depends on the presence of clonal plasma cells in the bone marrow or in a biopsy-proven bone or plasmacytoma (2, 15). Our patient presented with multiple vertebral compression fractures, anemia and clonal plasma cells in the bone marrow at diagnosis. Plasmacytoma, which has been described as a common extramedullary disease in patients aged younger than 20 years, did not occur in this case (16). The presence of an increased serum monoclonal component is uncommon in MM patients younger than 20 years of age (5, 13), while our patient showed a high serum monoclonal spike at diagnosis.

Regarding therapy, there has been a breakthrough in the treatment of MM over the past few years, which presently can rely on new therapies involving immunomodulators or proteasome inhibitors. According to the National Comprehensive Cancer Network, patients with MM should be treated including induction therapy with three drug regimens, including bortezomib/lenalidomide/dexamethasone, bortezomib/doxorubicin/dexamethasone, and bortezomib/cyclophosphamide/dexamethasone, plus hematopoietic stem cell transplantation (SCT) for patients under the age of 65 years who do not have substantial heart, lung, renal, or liver dysfunction (1, 2, 15). Although the majority of the population are not good candidates of ASCT in adults, ASCT after primary therapy has been standard of care of MM. Due to a limited information of pediatric MM, treatment strategies of this population are in line with adult patients. In a multicenter retrospective study of 52 patients diagnosed with MM before 30 years of age (age range, 8–30 years) showed the prognosis of MM in young patients was reported to be as good as if not better than that of general population of MM patients (15). This possibly due to the implementation of novel regimens and hematopoietic SCT in younger patients. On review of the literature, there were only few juvenile cases of pediatric MM who were treated with ASCT after induction therapy with good response (9, 10, 12). A case of an 8-year-old child diagnosed with MM achieved a very good partial response with a 92% reduction of the monoclonal component after ASCT (9). Two pediatric patients aged 12 and 16 years old with MM were treated with allogeneic SCT following autologous transplant. The 12-year-old patient recovered without progression of disease at 10-year follow-up, whereas the other passed away due to recurrent disease several months later after his second transplant (10). Another case of a 17-year-old girl with a stable clinical condition at 14-month follow-up indicates generic bortezomib based regimen coupled with SCT is a good treatment option for MM with low cost (12). Our patient started with induction therapy of bortezomib, cyclophosphamide, dexamethasone and rescued with ASCT. He obtained a good clinical response to treatment after bone marrow transplantation which was sustained for 5-month follow-up.

Although MM is rare in children and adolescents, more data regarding the efficacy of treatments in this population are needed. Treatment outcome of this case indicates that induction chemotherapy based regimen coupled with stem cell transplantation is a good treatment option for pediatric MM.

## Data Availability Statement

The original contributions presented in the study are included in the article/supplementary material. Further inquiries can be directed to the corresponding author.

## Ethics Statement

The studies involving human participants were reviewed and approved by the institutional review board in West China Hospital, Sichuan University. Written informed consent to participate in this study was provided by the participants’ legal guardian/next of kin. Written informed consent was obtained from the individual(s) for the publication of any potentially identifiable images or data included in this article.

## Author Contributions

XW collected the data and wrote the manuscript. HH and CL reviewed the literature and presented amendments for the paper. XW and MZ conducted the experiments. CJ edited and critically revised the manuscript. All authors contributed to the article and approved the submitted version.

## Funding

This work was supported by grant from the National Natural Science Foundation of China (81902142) and the Sichuan Science and Technology Department (2020YFS0096, 2020YFH0114).

## Conflict of Interest

The authors declare that the research was conducted in the absence of any commercial or financial relationships that could be construed as a potential conflict of interest.
